# The implementation, use and impact of patient reported outcome measures in value-based healthcare programmes: A scoping review

**DOI:** 10.1371/journal.pone.0290976

**Published:** 2023-12-06

**Authors:** Mayara Silveira Bianchim, Ellie Crane, Anwen Jones, Barbara Neukirchinger, Gareth Roberts, Leah Mclaughlin, Jane Noyes

**Affiliations:** 1 School of Medical and Health Sciences, Bangor University, Bangor, United Kingdom; 2 Aneurin Bevan University Health Board, Newport, United Kingdom; Iran University of Medical Sciences, ISLAMIC REPUBLIC OF IRAN

## Abstract

**Background:**

Value-Based Healthcare (VBHC) focuses on the value of patient outcomes and is achieved by ensuring resources already available are managed to realise the best possible individual and population health outcomes. Patient reported outcome measures (PROMs) measure the impact of illnesses from the patient perspective. We conducted a scoping review to understand how PROMs were implemented and used, and their impact in the context of VBHC.

**Methods:**

Arksey and O’Malley’s overarching framework supplemented by principles from mixed-methods Framework Synthesis were used. CINAHL, Cochrane Library, EMBASE, MEDLINE, PsycINFO, Web of Science, Google Scholar and reference lists were searched. An a priori data extraction framework was created using the review question and objectives as key domains against which to extract data. Mixed-methods data were organised, integrated and preserved in original format and reported for each domain.

**Results:**

Forty-three studies were included with 60,200 participants. Few studies reported a well-developed programme theory and we found little robust evidence of effect. PROMs were universally considered to have the potential to increase patient satisfaction with treatment and services, enhance patient awareness of symptoms and self-management, and improve health outcomes such as quality of life and global health status. Evidence is currently limited on how PROMs work and how best to optimally implement PROMs to achieve the target outcome. Implementation challenges commonly prevented the realisation of optimal outcomes and patients generally needed better and clearer communication about why PROMs were being given and how they could optimally be used to support their own self-management.

**Conclusion:**

PROMSs have yet to demonstrate their full potential in a VBHC context. Optimal PROMs implementation is poorly understood by clinicians and patients. Future studies should explore different models of PROM implementation and use within VBHC programmes to understand what works best and why for each specific context, condition, and population.

## Introduction

Value-Based Healthcare (VBHC) is a delivery model with the overarching goal of maximising value for patients and healthcare providers [1]. VBHC is achieved through the equitable, sustainable, and efficient use of resources to achieve better outcomes for every patient [1,2]. With growing demand being placed on finite health resources, the concept of VBHC has become increasingly important [2–4].

VBHC models are focused on patient-centred care, using outcomes that matter most to patients rather than relying solely on clinical measures [5,6]. Such metrics include mental and social functioning, health-related quality of life, disease symptoms and patient views on their health. Patient-reported outcome measures (PROMs) are a set of questions that seek to comprehensively capture these important metrics and are commonly used in research contexts [7]. PROMs are implemented within a VBHC setting with the aim of enabling healthcare providers to understand what matters most to patients, to better monitor, detect and if necessary, act-upon patient symptoms, and to facilitate shared patient-clinician decision making [7]. From the patient perspective, the aim of PROMs is to improve quality of care and health outcomes, improve patient understanding of their health, and promote active patient engagement with their own self-care and management [7].

PROMs have been established in healthcare for over a decade and are often an essential component in the delivery of person-centred care. However, there is a dearth of evidence on how to implement and use PROMs within a VBHC setting to maximise value for patients and health providers. Additionally, whether PROMs are effective in improving patient and health systems outcomes is also unclear. Addressing these questions is essential to help inform current and future PROMs interventions within a VBHC setting. Therefore, the aim of this scoping review was to identify and describe studies on the implementation, use and effectiveness of PROMs as part of a VBHC programme or a similar routine practice context.

## Material and methods

The methodology was guided by Arksey and O’Malley’s [8] five stage framework for scoping reviews:

- Stage 1: identifying the research question (i.e., defining the scope and review protocol)
- Stage 2: identifying relevant studies
- Stage 3: study selection
- Stage 4: charting the data
- Stage 5: collating, summarizing, and reporting the results

To manage and interpret a wide range of study designs, we incorporated principles of mixed-methods framework synthesis to extract, map, chart, categorise and aggregate study findings [9]. An a priori protocol was developed. In line with scoping review methodology, the level of synthesis was low with the output largely descriptive.

## Identifying the research question

A Setting, Perspective, Intervention/Phenomenon of Interest, Comparator, Evaluation (SPICE) framework was followed to structure the research question, objectives, and subsequent search strategy [10], as follows:

**Setting:** High income countries with similar health systems to the UK NHS. Primarily hospital based VBHC programmes that used PROMS.

**Perspectives:** Patients, carers, implementers, service providers, healthcare professionals, other key stakeholders. Any patient group or condition. In addition, we specifically looked at four diverse tracer services in greater depth:

- A surgical intervention (cataract surgery),
- A chronic disease with a large cohort of young adults (epilepsy),
- A chronic disease affecting a predominantly elderly and sometimes frail cohort (Parkinson’s disease), and
- A long-term chronic condition that is most common in older people but can affect people at any age (heart failure).

### Intervention/Phenomena of interest

1. What PROMs are used and what evidence is there that PROMs work?
2. How are PROMs used by patients, professionals, carers, the health service, and stakeholders?
3. How are PROMs intended to work to bring about specific outcomes?
4. How are PROMS implemented in four specific tracer conditions (cataract surgery, epilepsy, heart failure, Parkinson’s disease)?
5. What are the factors that create barriers and facilitators to PROMs implementation?
6. What (if any) are the unintended consequences of PROMs?
7. What are the experiences of patients and carers in using PROMs?
8. Are there differences in experiences or demographics across different services?
9. How are PROMs used with people (including family members and carers) with multiple co-morbid conditions?
10. Do PROMs raise any equity issues?
11. Are PROMs sustainable?
12. How translatable is this evidence?
13. What is the economic cost of developing or implementing PROMs programmes?

**Comparison:** Differences in experiences, perspectives and outcomes between groups and different ages, conditions, groups, contexts, ethnicity etc.

**Evaluation:** Scoping review to aggregate, describe and understand the evidence.

## Identifying relevant studies

The search protocol was developed and refined with the help of an expert librarian using a rigorous iterative process. Pilot searches were conducted to refine the search terms and assess the feasibility of the initial criteria. A systematic search for published studies was carried out in August to November 2022. The primary searches were conducted in CINAHL, Cochrane Library, EMBASE, MEDLINE, PsycINFO and Web of Science, and included relevant studies found via key word searches on Google Scholar. We also searched the VHBC study repository at a local health organisation. In addition, a non-comprehensive 3-word search targeting specific conditions was performed independently by two authors (MSB, EC), and each author used two different databases (PubMed and Google Scholar). The reference lists of all the identified systematic reviews were screened, with all potentially eligible studies subsequently assessed independently by two authors (MSB, EC) against the inclusion criteria.

The search was not designed to be exhaustive and was conducted iteratively in accordance with scoping review guidance [11]. A pilot search was performed to refine the Medical Subject Headlines (MeSH) terms and Boolean phrasing with the help of an experience librarian. The final search terms were inserted as keywords into all 9 databases were:

PROMS AND Patient Reported Outcome Measures AND VBHC AND Value Based Health Care AND Implementation Evaluation

## Study selection

We imported all searches to Mendeley (Elsevier, Amsterdam, Netherlands) for screening. Titles and abstracts of identified articles were screened by two people (EW, BC) independently to determine eligibility for inclusion. We included studies investigating the implementation, use, and impact of PROMs applied within the context of VBHC (i.e., the use of PROMs in healthcare to focus on outcomes that are important for patients, and/or used to increase value for patients and healthcare providers) (Table 1).

**Table 1:** Inclusion and exclusion criteria.

Inclusion	Exclusion
Full text peer-reviewed studies or grey literature	Abstracts or no full text available
Studies in the English language, unless a translation is readily available	Studies not available in English
PROMs used in a Value-Based Health Care, implementation study, service improvement or service evaluation setting.	Psychometric studies involving the development, validation, or reliability of PROMs
Studies in adult populations (>18 years)	Studies with children
Published after 2010 onwards	Studies published prior to 2010
Any methodology or design	Non-human or animal studies
Any clinical condition	

Full texts were retrieved and assessed independently by two authors (EW, MSB, BN, EC) against the eligibility criteria. Papers not meeting the inclusion criteria were excluded and the reasons for exclusion noted. Any disagreement between screeners was resolved by a third person until a consensus was reached.

## Charting the data

All papers were uploaded as PDF files and managed in Mendeley. A data extraction form which served as the a priori framework was developed using the phenomena of interest as key headings.

## Collating, summarizing, and reporting the results

An a priori data extraction framework was created using the review question and objectives as key domains against which to extract data. Using a process of familiarisation, studies were first marked up with notes and memos and key text of interest highlighted and then extracted into the a priori framework on an excel spreadsheet (S1 Table). Supplementary information for each study was obtained where available and when necessary primary study authors were consulted to obtain or confirm data. Having extracted all data of interest into the framework, mapping and charting was undertaken to visualise and interpret each element of interest. PROMs were first viewed as a cross-disciplinary general intervention. Mixed-methods data were organised, integrated and preserved in original format and reported for each domain in the a priori framework that corresponded to the review question and objectives. Then evidence was sought and configured on PROMs specifically for the four tracer conditions. Through this process we developed descriptive level findings and explanations. Findings were shared and discussed with a wider group of researchers and discussed with key stakeholders. The review was reported using the relevant domains of the Preferred Reporting items for Systematic Review and Meta-Analysis for scoping reviews (PRISMA-ScR) (S2 Table) [12].

### Quality assessment

All included studies were independently appraised by two reviewers (AJ, BN, EC, GR, JN, LM, MSB) using the Quality Assessment for Diverse Studies (QuADS) tool [13]. The tool was designed to appraise mixed or multi-methods studies in complex systematic reviews in health services research. The QuADS tool [12] is reported to demonstrate strong inter-rater reliability (k = 0.66), and substantial content validity, and is composed of 13 domains [12]. Two reviewers (EC & MSB) piloted the tool on five studies encompassing different designs prior to assessment.

The checklist usually includes a final score for quality assessment, which we did not calculate. This is because total quality scores are considered unhelpful as the domains assessed do not impact equally on the quality of the study. What is more important is the identification of methodological limitations in primary studies and how these limitations may impact on the interpretation of findings [14]. We used the checklist to assess the level of methodological concerns rather than calculate a total quality numeric score. All studies were appraised according to the level of methodological concern: ‘no/minor’, ‘moderate’, ‘serious’, or ‘very serious’ concerns. Studies were not excluded based on their methodological limitations, but findings from studies with serious and very serious methodological concerns were interpreted with caution. All disagreements were discussed and resolved, and a third review author was consulted when necessary. All assessments were transparently recorded using Microsoft Excel.

### Stakeholder engagement

Stakeholders with experience using PROMs as health care professionals or working with relevant health conditions, staff working in relevant third sector organisations and established stakeholder and patient advocacy groups were invited to participate in engagement sessions (i.e., St. David’s Hospice Care, British Heart Foundation, Digital Wales, Epilepsy Action, Digital Communities Wales, Parkinson’s UK Cymry, Race Equality First, Aneurin Bevan Community Health Council and VBHC Patient Reference Group). Engagement sessions with stakeholders were planned strategically and the discussions were tailored for each group according to their background and lived experience. Stakeholder input was primarily used to provide context and inform the interpretation of findings and help identify gaps in evidence. For example, stakeholder engagement helped with the interpretation of facilitation and barriers factors for PROM implementation, disease-specific aspects of PROMs and digital literary and issues related to equality, inclusion and diversity.

## Results

**Fig 1:**
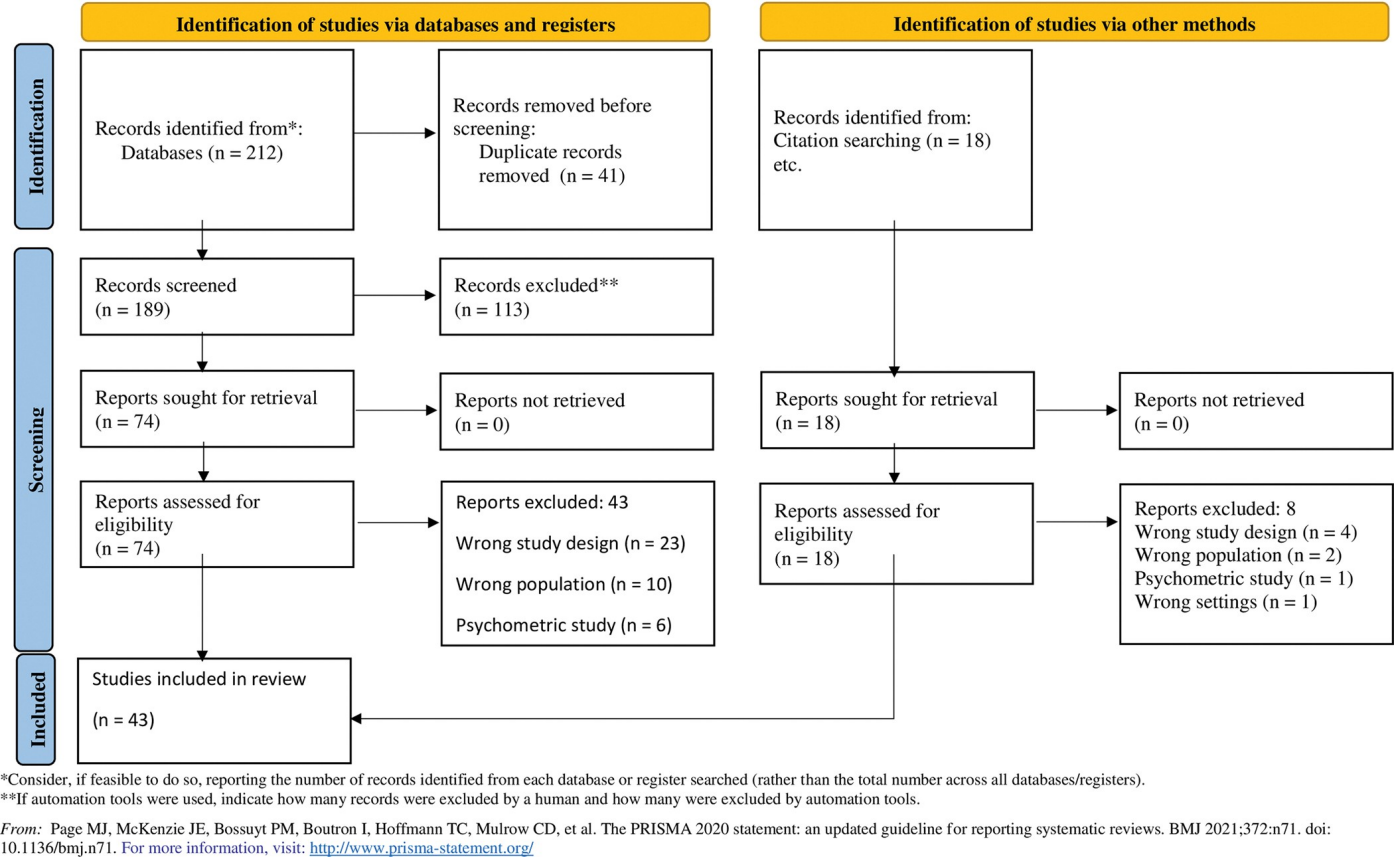
PRISMA flow-chart.

Forty-three studies were included in total. Among these, 39 studies reported a total of 60,200 participants aged between 18 to 103 years; and 31 studies reported that 56.8% of participants were female (n = 18,845) (Fig 1 and S3 Table). Included studies investigated various PROMs interventions, across 13 countries, and across a wide range of conditions (Table 2). Twenty-four studies specified investigating the use of PROMs specifically in a VBHC program [15–38], while the other 19 studies [39–56], investigated aspects of PROMs implementation in routine practice that were relevant to our research questions (language barriers, multiple comorbidities, tracer conditions etc.).

**Table 2:** Patient Reported Outcome Measures [PROMs] per condition and response rates [n = 39 studies].

Health Condition / Topic	Author & year	PROMs used	PROM delivery method	Response rates [%]
Asthma (n = 4)	Peters et al (2013) [30] Peters et al (2014) [31] Peters et al & Croker et al (2014) [32]	Generic: • EuroQoL EQ-5D Disease specific: • Mini Asthma Quality of Life Questionnaire (mini-AQOL)	Paper questionnaire delivered by post	30.0%
	Porter et al (2021) [50]	Generic: • EuroQoL EQ-5D • Patient Generated Index (PGI) Disease specific: • Mini Asthma Quality of Life Questionnaire (mini-AQOL)	Delivered in general practice. Specific method not provided	100%
Cancer (n = 8)	Ashley et al (2013) [41]	Illness Perception Questionnaire-Revised EuroQol-5D, Version 2 Medical Outcomes Study 36-Item Short-Form Health Survey, Version 2 Social Difficulties Inventory European Organisation for Research and Treatment of Cancer Quality of Life Questionnaire Quality of Life in Adult Cancer Survivors Scale	Digitally	55.21% overall, 61.4% face-to-face, 48.8% over the phone, 41% via letter
	Basch et al (2016) [42]	PROM questionnaire adapted from the National Cancer Institute’s Common Terminology Criteria for Adverse Events regarding 12 common symptoms reported during chemotherapy	Digitally	73%
	Demedts et al (2021) [18]	EORTC Core Quality of Life questionnaire (EORTC QLQ-C30) EORTC QLQ-LC13: A 13-item lung cancer-specific questionnaire	Digitally	92%
	Nguyen et al (2019) [25]	The European Organisation for Research and Treatment of Cancer Quality of Life questionnaire (EORTC QLQ-C30)	Paper questionnaire	100% at baseline, 93.8% during therapy, 100% at the end of therapy and 100, 85.7, 83.3 and 66.7% every 3 months until 1 year after therapy, respectively
	Schuler et al (2017) [52]	EuroQoL EQ-5D The European Organisation for Research and Treatment of Cancer Quality of Life questionnaire (EORTC QLQ-C30)	Digitally	34.2% at admission and 17.3% at discharge
	van Egdom et al (2019) [37]	The European Organisation for Research and Treatment of Cancer Quality of Life questionnaire (EORTC QLQ-C30) The European Organisation for Research and Treatment of Cancer Quality of Life for Breast Cancer (EORTC-QLQ B23) BREAST-Q pre-operative and post-operative modules EQ-5D-5L Distress Thermometer The Reproductive Concerns Scale (RCS-NL) The CarerQoL-7D	Paper questionnaires	83.3% at baseline, 65.7% at 6 months and 55.1% at 12 months
	Wheelock et al (2015) [55]	Short Form Health Survey (SF-36) Personal Health Questionnaire Depression Scale (PHQ-8) Symptom questions modified from the Memorial Symptom Assessment Scale	Digital	Not reported
Cataract Surgery (n = 7 studies)	Devlin et al (2010) [19]	EuroQoL EQ-5D The Visual Focus Index 14 (VF-14)	Paper questionnaire	Not reported
	Fung et al (2016) [45]	EuroQoL EQ-5D EQ-VAS visual analogue scale National Eye Institute Socioemotional Scale (NEI-SES) The Short-form Visual Function Index (VF-8R)	Paper questionnaire delivered by post	67.2% at 3 weeks after surgery, 61.8% at 3 months after surgery. 30% non-response rate
	Queirós et al (2021) [33]	CATQUEST-9SF	Paper questionnaire in clinic	Not reported
	Sparrow et al (2018) [53]	CATQUEST-9SF CAT-PROM5	Not reported	Not reported
	Sparrow et al (2020) [35]	CAT-PROM5	Digitally	94.3% at pre-operative time point and 36.4% post-operative
	Tognetto et al (2021) [54]	CATQUEST-9SF	Not provided	Not reported
	Zijlmans et al (2021) [56]	CATQUEST-9SF	Not provided	Not reported
Chronic Obstructive Pulmonary Disease (COPD) (n = 4)	Peters et al (2013) [30] Peters et al (2014) [31] Peters et al & Croker et al (2014) [32]	Generic: • EuroQoL EQ-5D Disease specific: • Clinical COPD questionnaire (CCQ)	Paper questionnaire delivered by post	49.2%
	Porter et al (2021) [50]	Generic: • EuroQoL EQ-5D • Patient Generated Index (PGI) Disease specific: • Clinical COPD Questionnaire (CCQ) • MRC breathlessness scale	Delivered in general practice. Specific method not provided	100%
Diabetes (n = 5 studies)	Peters et al (2013) [30] Peters et al (2014) [31] Peters et al & Croker et al (2014) [32]	Generic: • EuroQoL EQ-5D Disease specific: • The Diabetes Health Profile (DHP)	Paper questionnaire delivered by post	40%
	Porter et al (2021) [50]	Generic: • EuroQoL EQ-5D • Patient Generated Index (PGI) Disease specific: • The Diabetes Health Profile (DHP)	Delivered in general practice. Specific method not provided	100%
Epilepsy (n = 8 studies)	Clary et al (2022) [43]	QOLIE-10 Generalized Anxiety Disorder (GAD-7) scale Neurological Disorders Depression Inventory for Epilepsy (NDDI-E)	Telephone or online via electronic health records portal	66.7% for 6 months for patients using electronic health record and 100% for telephone PROMs collection
	Moura & Magliocco et al (2016) [24]	Patient-Reported Outcome Measurement Information System–10 (PROMIS-10) Quality of Life in Epilepsy ii Inventory (QOLIE-31)	Digitally in clinic	49.3%
	Moura & Schwamm et al (2019) [49]	Newly developed questionnaire for medication adherence & side-effects, seizure frequency, and driving. This questionnaire included the Patient-Reported Outcome Measurement Information System–10 (PROMIS-10) measure	Digitally in clinic	44.8% at epilepsy clinic. Response rates were 12.3%, 51.1%, and 36.6 for the first, second, and third months of data collection, respectively
	Peters et al (2013) [30] Peters et al (2014) [31] Peters & Croker et al (2014) [32]	Generic: • EuroQoL EQ-5D Disease specific: • Quality of Life in Epilepsy ii Inventory (QOLIE-31)	Paper questionnaire delivered by post	34%
	Sajobi et al (2021) [51]	Quality of Life in Epilepsy (QOLIE10-P) measure. Epilepsy Comorbidity Index (for depression and anxiety)	Not provided	Prospective data from the Calgary Comprehensive Epilepsy Program
Heart Failure (n = 7 studies)	Kane et al & Daveson (2017) [47] Kane & Ellis-smith et al et al (2017) [47]	Kansas City Cardiomyopathy Questionnaire (KCCQ) Patient Health Questionnaire-8 (PHQ-8) A quality-of-life visual analogue scale	Telephone questionnaire	66%
	Pennucci et al (2020) [29]	Kansas City Cardiomyopathy Questionnaire-12 (KCCQ-12) Self-Care Heart Failure Index (SCHFI) (Italian translation)	Questionnaire by phone or email	64% at baseline, 61% at 1 month, 49% at 7 months and 31% at 12 months. Response rate was higher when patients gave only a caregiver contact (80% vs 64.2%)
	Peters et al (2013) [30] Peters et al (2014) [31] Peters et al & Croker (2014) [32]	Generic: • EuroQoL EQ-5D Disease specific: • Minnesota Living with Heart Failure Questionnaire (MLHFQ)	Paper questionnaire delivered by post	50%
	Porter et al (2021) [50]	Generic: • EuroQoL EQ-5D • Patient Generated Index (PGI) Disease specific: • Minnesota Living with Heart Failure Questionnaire (MLHFQ)	Delivered in general practice. Specific method not provided	100%
Orthopaedic Conditions (n = 5 studies)	Bernstein et al (2019) [61]	The Patient‐Reported Outcomes Measurement Information System (PROMIS) questionnaire including items on physical function, pain interference, and depression	Digitally in-clinic	Not reported
	Devlin et al (2010) [19]	EuroQoL EQ-5D Oxford Knee Score (for knee replacements) Oxford Hip Score (for hip replacements) Short form heath survey (for hip replacements)	Paper questionnaire	92% for hip replacement
	Liu et al (2018) [57]	PROMIS Global Health measure Hip Disability and Osteoarthritis Outcome Score Knee Injury and Osteoarthritis Outcome Score	In person paper questionnaire	Not reported
	Malhotra et al (2016) [48]	EuroQoL EQ-5D EQ-VAS visual analogue scale	Digital	85.9%
	Papuga et al (2017) [28]	PROMIS computer adaptive test (CAT) instruments: • Physical function • Pain interference • Depression	Digitally	Not reported
	Porter et al (2021) [50]	Generic: • EuroQoL EQ-5D • Patient Generated Index (PGI) Disease specific: • Oxford Hip Score (for knee replacements) • Oxford Knee Score (for knee replacements)	Delivered in general practice. Specific method not provided	100%
Stroke (n = 5 studies)	Groeneveld et al (2019) [21]	EuroQoL EQ-5D Stroke Impact Scale (SIS) Stroke and Aphasia Quality of Life Scale (SAQOL-39NL) HADS Utrecht Scale for Evaluation of Rehabilitation-Participation (USER-P) Fatigue Severity Scale (FSS)	Paper or digital	60% response rates for inpatients and 43.3% response rates for outpatients
	Oemrawsingh et al (2019) [27]	EuroQoL EQ-5D	Telephone or in person interviews	Prospective data
	Peters et al (2013) [30] Peters et al (2014) [31] Peters et al & Croker (2014) [32]	Generic: • EuroQoL EQ-5D Disease specific: • Stroke Impact Scale (SIS)	Paper questionnaire delivered by post	36.4%
Varicose vein surgery Groin hernia repair	Devlin et al (2010) [19]	EuroQoL EQ-5D Short form heath survey (SF-36) (for groin hernia repair) Aberdeen Varicose Vein Symptom Severity questionnaire (for varicose vein surgery)	Paper questionnaire	75%
Bariatric surgery	Goretti et al (2020) [20]	Bariatric Analysis and Reporting Outcome System (BAROS) Questionnaire for physical activity, work capability, dressing, and sexual activity	In person interview by clinicians	82% response rate at follow-up (phone calls), and 83.4% seven days and 1-year follow-up after surgery
Pregnancy & childbirth	Laureij et al (2020) [22]	Patient-Reported Outcome Measurement Information System–10 (PROMIS-10) to track perceived quality of life. Depression during pregnancy or postpartum, screened with Patient Health Questionnaire-2 (PHQ-2) Breastfeeding Self-Efficacy Scale-Short Form (BSES-SF) International Consultation on Incontinence Questionnaire-Short Form (ICIQ-SF) or PROMIS SFFAC102 to measure incontinence and pain with intercourse Mother-Infant Bonding Scale (MIBS) Birth Satisfaction Scale-Revised (BBS-R)	Digitally	39%
Advanced chronic kidney disease (CKD)	van der Willik et al (2019) [36]	PROMs questionnaire developed for chronic kidney disease (CKD) symptoms	Digitally	Not reported
Implementation of PROMs tool for wide range of conditions	O’Connell et al (2018) [26]	Developed generic PROM tool with three components: • The EQ-5D-5L questionnaire • The Work Productivity and Activity Impairment (WPAI) tool • ’About You’ questions on height, weight smoking history, exercise levels, alcohol consumption and medically diagnosed comorbidities	Digitally	Not reported
	Rutherford et al (2021) [34]	Patient-Reported Outcome Measurement Information System–10 (PROMIS-10) Depression Anxiety Stress Scales (DASS21) (only available at specific sites) Chronic obstructive pulmonary disease Assessment Test (CAT) (only available at specific sites)	Digitally	69% at baseline and 55.6% at follow-up

### Methodological strengths and limitations of included studies

The majority of included studies were judged to have no or minor methodological concerns 79% (n = 33), followed by 14% (n = 6) moderate methodological concerns and 7% (n = 3) serious methodological concerns. No study was judged to have very serious methodological concerns (S4 Table). For most studies, methodological concerns were due to a lack of reporting rather than methodological limitations. For example, the lack of recruitment information was the second most common limitation encountered. The main limitation encountered was the absence of stakeholder involvement in research design or conduct. Data collection and analysis were mostly well designed and conducted across the studies.

### Factors that created barriers and facilitators to optimal implementation

Thirty-one studies described factors that created barriers and facilitators to PROMs implementation. Many of the factors described were bi-directional, acting as either facilitators or barriers depending on the context and whether the factor was present or not. We identified four groups of factors in the implementation of PROMs (Table 3). These groups included digital and technology factors, factors associated with patients and carers, factors associated with healthcare staff and stakeholders, and structural & organisational factors.

**Table 3:** Factors that created barriers and facilitators at different stages of PROMs implementation.

	Preparation for implementation	Implementation in practice	Sustainability in the long term
**Digital and technology factors**	Electronic PROM systems that are integrated with patient medical records* [15,18,22,26,29,30,34,35,37–40,49,53] IT support staff^#^ [18,34,35,37,38] Costs for software and digital equipment such as tablets, computers, software etc^ø^ [24,39,49]	Reliable internet [18,34,35,37,38] Electronic PROMs systems that are integrated with patient medical records* [15,18,22,26,29,30,34,35,37–40,49,53] Accessible and well-functioning digital systems that require limited effort from clinical staff with data collection, analysis, and reporting* [15,22,29,34,38,39,49] Automated PROMs pathways* [18,38,42,49] IT support staff^#^ [18,34,35,37,38]	Reliable internet [18,34,35,37,38]. Electronic PROMs systems that are integrated with patient medical records* [15,18,22,26,29,30,34,35,37–40,49,53] Accessible and well-functioning digital systems that require limited effort from clinical staff with data collection, analysis, and reporting* [15,22,29,34,38,39,49] Automated PROMs pathways* [18,38,42,49] IT support staff^#^ [18,34,35,37,38]
**Factors associated with patients & carers**	Planning for dedicated time to complete PROMs for patients^#^ [15,34,39,46] Planning for hybrid delivery [digital / paper PROM] to allow for patient preference and requirements, and to improve retention^#^ [8,12,24,27,28] Planning provisions for patient with poor language proficiency in the main healthcare language, particularly in multicultural locations^#^ [22,34,39,49] Carefully developing PROM content with stakeholder engagement to ensure it is acceptable and feasible to target population i.e., not too long, well explained, understandable, captures what is important^#^ [15,30,34,46,50]	Providing dedicated time to complete PROMs for patients^#^ [15,34,39,46] Length and difficulty to complete PROMs^ø^ [15,30,34,46,50] Caregivers helped patients with language, technology, or physical/mental impairment barriers, resulting in improved accessibility of PROMs to often excluded groups]^#^ [4,11,13,16–20,26] Patient not understanding the content of the PROMs questions or becoming upset over being confronted by their condition^ø^ [6,8,15] Poor patient understanding about what PROMs are and how they are used in their healthcare^ø^ Clear communication about PROMS with patients and carers is very important^#^ [15,30,34,37,46] Digital literacy, particularly for patients with cognitive impairments^ø^ [18,34,37,41,46,50] Hybrid delivery [digital / paper PROM] to allow for patient preference and requirements, and to improve retention^#^ [8,12,24,27,28] Digital literacy^ø^ [18,21,34,37,41,52] Reminders to complete PROMs^#^ [29,38,58] Physical and mental health impairments^ø^ [25,29,46] Poor language proficiency in the main healthcare language^ø^ [22,34,39,49]	Providing dedicated time to complete PROMs for patients^#^ [15,34,39,46] Caregivers helped patients with language, technology, or physical/mental impairment barriers, resulting in improved accessibility of PROMs to often excluded groups^#^ [4,11,13,16–20,26] Patient understanding about what PROMs are and how they are used in their healthcare^ø^ Clear communication about PROMS with patients and carers is very important^#^ [15,30,34,37,46] Digital literacy, particularly for patients with cognitive impairments^ø^ [18,34,37,41,46,50]. Hybrid delivery [digital / paper PROM] to allow for patient preference and requirements, and to improve retention^#^ [8,12,24,27,28]. Poor language proficiency in the main healthcare language^ø^ [22,34,39,49] Reminders to complete PROMs^#^ [29,38,58] Digital literacy^ø^ [18,21,34,37,41,52] Physical and mental health impairments^ø^ [25,29,46]
**Factors associated with healthcare staff & stakeholders**	Leadership and staff resistance^ø^ [30,34,35,39] Management of staff capacity and responsibility in relation to the additional clinical burden of PROMs* [6,8,15,24,27,30,33] Provision of dedicated PROMs support staff^#^ [8,12,17] Staff motivation, engagement and ownership in implementation and delivery of PROMs* [22,30,34,35,37,44,59] Staff training and support for clinicians and staff. This is essential in ensuring PROMs are implemented as intended and that staff understand the purpose of PROMs, helping to consolidate engagement. It also provides space for staff to voice concerns and find collaborative solutions* [17,27–29,34,35,38,49,53,59,61]	Leadership and staff resistance^ø^ [30,34,35,39] Management of staff capacity and responsibility in relation to the additional clinical burden of PROMs* [6,8,15,24,27,30,33] Provision of dedicated PROMs support staff^#^ [8,12,17] Disruption to clinical flow^ø^ [27,30,34] Ongoing staff training and support for clinicians and staff* [17,27–29,34,35,38,49,53,59,61] Staff motivation, engagement, and ownership in delivery of PROMs^#^ [22,30,34,35,37,44,59]	Leadership and staff resistance^ø^ [30,34,35,39] Staff ownership, teamwork, and collaboration* [22,30,34,35,37,44,59] Staff understanding of PROMs* Provision of dedicated PROMs support staff^#^ [8,12,17] Administrative assistance for clinical staff^#^ Ongoing staff training and support for clinicians and staff* [17,27–29,34,35,38,49,53,59,61] Staff motivation, engagement, and ownership in delivery of PROMs^#^ [22,30,34,35,37,44,59] Disruption to clinical flow^ø^ [27,30,34]
**Structural and organisational factors**	System wide institutional support [managerial, IT, financial]* [24,38,39,49] Well thought through planning, incorporating engagement with key stakeholders at all stages^#^ [24,29,34,42,52] Availability of multilingual valid translated PROMs^#^ [39,49] System wide implementation can be more efficient in terms of scalability and costs^#^ [22,38] Communication within and between services^#^ [15,38,49] Dedicated time and resources to implement and deliver PROMs^#^ [15,29,34]	Resource availability [staff, digital, financial]* [15,24,34,35] System wide institutional support [managerial, IT, financial]* [24,38,39,49] Well thought through delivery, incorporating engagement with key stakeholders at all stages^#^ [24,29,34,42,52] Ongoing evaluation and iterative refinement of PROMs systems. Small incremental changes may be a better approach^#^ [37,38] Communication within and between services^#^ [15,38,49]	Resources availability [staff, digital, financial]* [15,24,34,35] System wide institutional support [managerial, IT, financial]* [24,38,39,49] Ongoing evaluation and iterative refinement of PROMs systems^#^ Small incremental changes may be a better approach. Stakeholders should be incorporated^#^ [37,38] Communication within and between services^#^ [15,38,49] Data management capacity^#^ [27] Flexibility to change over time^#^ [21] Co-production design^#^ [37]

^#^Predominantly facilitator.

*Bidirectional, can be both a barrier and facilitator.

^ø^Predominately barrier.

### Programme theory

We have identified two main programme theories explaining the mechanisms by which PROMs were thought to improve patient outcomes. These theories are not mutually exclusive, and analysis of included studies suggested multiple mechanistic pathways associated with PROM interventions.

#### Theory 1: PROMs promote proactive communication and positive health behaviours in patients

One possible mechanism is that by completing PROMs patients were prompted to reflect on their symptom, thereby improving awareness of their health and wellbeing. PROMs helped to validate patients’ concerns and empowered them to raise these issues with clinicians, thus improving patient-clinician communication. Additionally, enhanced patient awareness regarding their own health potentially increased their engagement in positive health-related behaviours [15,17,21,23,29–31,33]. We found evidence that PROMs promoted self-reflection [18,24,46,49], helped patients to identify their needs and priorities [18,34,46,49], and promoted more active engagement from patients in managing their own health [18,34,46,50,55].

#### Theory 2: PROMs increase clinician awareness of patient symptoms

PROMs provided regular feedback to clinicians highlighting undetected issues or symptoms, and/or changes in symptoms. Improved symptom detection subsequently enhanced the quality of appointments and benefited patient health outcomes [38,42,49]. Better symptom awareness and detection promoted quicker treatment and tailoring of care according to the needs of patients [13,15,17,19,29,37]. Clinicians reported that PROMs enabled them to prioritise topics for discussion during appointments, which resulted in better shared decision-making [18,37,38,42,46,49,50,52].

### Effectiveness of PROMs interventions

#### Health outcomes

Two studies showed statistically significant improvements in health outcomes in cancer patients as a result of a VBHC PROM intervention [18,42]. In these studies, PROMs data was collected regularly and used to automatically alert the healthcare team when a predefined threshold indicated need of clinical attention [18,42]. Patients receiving the PROMs had higher survival, a lower decrease in health-related quality of life and remained on chemotherapy for longer compared to the treatment-as-usual group [18,42]. Additionally, patients receiving PROMs also had less emergency care visits, were less frequently hospitalised, and had shorter lengths of stay in clinic compared to those in usual care [18,42,23,29,42].

Patient health-related behaviours. Several studies demonstrated that PROMs positively influenced health-related behaviours in patients, such as symptom reporting and more active engagement in their own healthcare and management.

#### Symptom reporting

Patients on cancer treatment completing PROMs were more likely to report symptoms compared to those in usual care, particularly for symptoms not perceived as urgent by the patient [55]. PROMs also helped patients with heart-failure to raise health-related problems with their clinician [46]. Specifically, patients described that PROMs provided the language to explain these issues and validated problems as worthy of reporting. PROMs also helped patients to raise symptoms associated with stigma such as pelvic dysfunction or mental health problems [22,46].

#### Improved patient health management

PROMs helped patients to actively engage in managing their own health [18,34,46,50,55]. Completing PROMs improved patient awareness of their everyday functioning and of own health [37,41,46,52], which helped them take ownership of managing their symptoms [46,47]. PROMs also helped patients prepare for appointments and facilitated communication with clinicians [37,52].

#### Patient perspectives on PROMs

The response rate of PROMs completion varied from 30% to 100%. The lowest response rate was seen in asthma while diabetes, orthopaedic conditions and chronic obstructive pulmonary disease (COPD) had the highest response rates.

Seven studies reported that patients found PROMs helpful and using PROMs improved their quality of care [18,22,34,37,46,47,60]. More specifically, Porter [50] reported that 92% of patients agreed that PROMs were easy to understand and helped during clinical appointments, and 76% would like PROMs to be included as part of their routine care [50]. In contrast, four studies reported that PROMs were not helpful, were overly bureaucratic, a waste of resources, more for the benefit of clinicians/researchers than patients, and that they did not adequately capture symptoms also voiced more critical patient perspectives regarding PROMs [30,46,48,50].

#### Impact of PROMs on healthcare professionals and clinical practice

Eleven studies reported clinicians used patient-reported data to better detect health problems, and tailor treatment to the most appropriate care and support provision [18,24,29,34,37,38,40,42,46,49,52,55]. PROMs were also used in clinical care as a triage tool to signpost patients to the right service at the right time [34]. Additionally, several healthcare professionals reported that PROMs enabled feedback of patient health status between appointments [37,38,42,47,46,49]. Automated PROM systems allowed both clinicians and patients to objectively track changes in health status and mental health over time without an increase in workload [24,29,38,42,49].

#### PROMs and service monitoring

Ten studies described PROMs helped to critically appraise, evaluate, and improve service provision to better meet patient and staff needs [21,33,34,38,56]. This often entailed using longitudinal PROMs data to track, inform, and refine services [21,24,29,34,37,38,49], leading to improved efficiency, better management of resources, and improved patient care [21,24,29,33,34,37,38,49]. For instance, a VBHC service in Wales used longitudinal PROM data to inform high-level decision making, which resulted in continued improvement of services [38].

### PROMs use with multiple co-morbid conditions

There was a lack of evidence investigating the use of PROMs in patients with multiple comorbidities. Patients with comorbid conditions were typically required to complete several PROMs for each condition, which was perceived as time consuming and repetitive [50]. With some notable exceptions, there was little linking across the PROMs used by the various services. Porter [50] combined PROM administration to patients with co-morbidities to reduce the overall number of PROMs that patients had to complete and avoid duplicate questions]. Additionally, Withers [17] noted the importance of electronic systems to allow the integration of multiple PROM pathways for patients with co-morbidities.

### Transferability and generalisability

Thirteen studies were large scale with sample sizes ranging from 822 to 17,892 participants [19,20,26–28,30–32,34,48,49,51,53,56,60], and fourteen studies evaluated the use of PROMs in more than one centre [17,19,21,22,26,30–32,34,38,40,41,46,47,53,56]. Studies evaluated the use of PROMs across 26 health care conditions. Factors that limited transferability included studies conducted in single healthcare centres [15,18,20,28,37,42,43,45,52,54,55,60], the variety different health care models [e.g., private healthcare], and the prominence of studies conducted in academic hospitals that may not be sufficiently similar to hospitals not associated with academic institutions (e.g. resources, staff patient ratios etc) [15,17,20,27,28,37,39,40,52,54,55,60]. It cannot therefore be assumed that the results of these studies will extrapolate to global practice.

### Cost-effectiveness

We found limited evidence to inform the current understanding on the cost effectiveness of PROMs interventions [13]. PROMs interventions were reported to potentially reduce the need of resources indirectly as it resulted in a reduction of length of hospital stay, emergency department visits and hospitalisations [18,42]. However, not all studies found a significant reduction in appointments and medical tests between patients receiving PROMs compared to patients receiving standard care [55].

### Tracer conditions

Configuring the evidence for the four tracer conditions did not add anything to our overall understanding. For completeness, we present the studies organised by the four tracer conditions in S1 File.

## Discussion

We found 43 diverse study designs investigating the implementation, use and impact of PROMs in a broad range of disciplines and specialities. Although there were some descriptions of how PROMs were intended to work, few studies reported a well-developed programme theory. With some notable exceptions (such as early identification of symptoms in cancer), we found little robust evidence of the effectiveness of PROMs. PROMs were universally considered to have the potential to increase patient satisfaction with treatment and services, enhance patient awareness of symptoms and self-management, and improve health outcomes such as quality of life and global health status. PROMS were generally seen by patients as providing information for healthcare professionals. Implementation issues commonly prevented the realisation of optimal outcomes and patents generally needed better and clearer communication about why PROMs were being given and how they could optimally be used by patients to support their own self-management.

Beyond a VBHC context, a Cochrane review [61] including 116 randomised controlled trials that specifically included PROMs feedback as part of the intervention in a broader range of settings and contexts found moderate evidence, calculated as measures of treatment effect size, that PROM feedback improved quality of life, and increased patient-physician communication, and disease control. However, this review also highlights the uncertainty regarding the impact of PROMs on general health perception, pain, fatigue, and on physical, mental, and social functioning [61]. In addition to the benefits associated with PROMs feedback, our scoping review suggested that PROMs longitudinal data helped to evaluate health services and even led to updated models of service delivery. This is supported by the review by Gibbons [61], which demonstrated that PROMs data facilitated quality improvement of services and were regarded as having substantial value beyond informing treatment. This corroborated our finding that PROMs in VBHC can help to evaluate the provision of healthcare and identify issues for improvement and inform the change within existing care pathways when necessary. However, evidence of real-world PROMs implementation and specifically within a VBHC programme is still limited [62], or when available, aggregated PROMs data seemed to be scarcely used to tailor treatments or improve services [44]. For instance, a recent review [63] reported little to no effect of aggregated PROM data on quality improvement methods in healthcare and highlighted the need for more empirical research. Bureaucratic challenges and the accessibility of IT systems integrated within current electronic health records was the main barrier to optimal implementation and use of PROMs data identified in this review. This finding is widely supported by other reviews [16,44,63–68]. For example, Gensheimer [65] recommended that PROMs integration into electronic health records is context-dependent and should be guided by multidisciplinary expertise to balance the advantages and disadvantages for each service [61].

### Strengths and limitations

An a priori protocol was developed, and the scoping review was conducted using systematic processes. The incorporation of different research designs and methods is particularly relevant in health care research considering the complexity of some aspects of health that cannot be readily quantified (e.g. lived experiences) [69]. The broad focus enabled a comprehensive understanding of the use, implementation, and impact of PROMs within a VBHC setting involving a multidisciplinary team of seven core researchers. It is not a requirement to assess methodological strengths and limitations of included studies in scoping reviews, but we elected to do so.

Some limitations are worthy of note. Due to time constraints, the search strategy was not exhaustive. Therefore, some papers eligible for inclusion may not have been identified. Despite that, a considerable number of databases were searched, and a strategic 3-word search was also conducted. As this is a scoping review, we aimed to provide a broad overview on the use of PROMs within a VBHC or broadly similar setting. While this allowed us to have a detailed overview of the evidence, we had to compromise on depth and specificity. There may be additional useful evidence of PROMs use outside of VBHC programmes to further enhance understanding [13].

### Gaps and future research

Evidence about how PROMs work and how best to implement and deliver PROM interventions to optimise achievement of the target outcome within a VBCH and routine practice setting is currently limited. The routine practice and VBHC context are quite different to a time limited research context whereby patients usually complete a set number of PROMs over a defined period of time. It is clear that PROMs do not consistently translate from short-term research to a long-term routine practice context and we need to understand why in order to address the implementation, feasibility and acceptability issues.

More empirical evidence is needed to demonstrate the value of PROMs and the benefits to services and patients. Whilst there is a growing number of implementation, feasibility and pilot studies, there is a lack of large-scale randomised controlled trials (RCTs) evaluating PROMs in a VBHC setting. A recent Cochrane review [61] included RCTs where PROMs were used for evaluation rather than the PROMs being the intervention. RCTs are however expensive and may not be the best way of evaluating PROMs in real world contexts as part of a complex intervention in a complex health system. Addressing these gaps in evidence is critical to help inform future strategies regarding the selection, implementation and use of PROMs by patients, carers and healthcare professionals as part of a VBHC programme in routine practice settings. VBHC programmes using PROMs are expensive and time consuming for patients and health care professionals to use. PROMs need to work better and be more highly valued in order to become a long-term sustainable component of routine practice.

More research is needed evaluating the impact of sustained implementation, delivery and costs of PROMs within a healthcare service to understand the full potential of PROMs in clinical practice. We need more understanding of how the proposed theoretical mechanisms of PROMs work in practice. We also found a gap in the evidence about how disease-specific factors might impact the implementation and use of PROMs, which is particularly important for patients with multiple conditions. Indeed, no new findings were highlighted when we configured and analysed the evidence for the four tracer conditions. Further research should investigate the impact of disease-specific factors in the implementation and use of PROMs, particularly in patients with multiple comorbidities. Additionally, building a broader evidence-base evaluating different models of PROM interventions is needed to understand what works best for which conditions, healthcare settings and populations. This is essential for the future developments of evidenced-based, best-practice guidelines for PROMs. Few studies investigated the role of caregivers in health care management [29,60]. Where appropriate, future research should address whether PROMs are feasible and acceptable to caregivers and incorporate caregivers into the design and delivery of PROM interventions. Future studies would also benefit from more integrated stakeholder and patient and public involvement when developing and implementing PROMs in order to capture what is important to patients and healthcare providers. We have subsequently embarked on a large scale realist evaluation and social return on investment analysis to address some of the identified gaps to further support optimal implementation of PROMs in VBHC programmes.

## Conclusion

This scoping review has mapped and described what is known and current evidence gaps and sets out a future research agenda. Value-Based healthcare programmes are being rolled out at scale in many different health systems and contexts. PROMs are commonly used in VBHC programmes but they have yet to demonstrate their full potential in a VBHC context. Optimal PROMs implementation is poorly understood by clinicians and patients.

## Supporting information

S1 Table Framework. Framework used for data analysis.
(DOCX)

S2 Table PRISMA checklist results. (DOCX)

S3 Table Characteristics of included studies. Characteristics of 43 studies.
(DOCX)

S4 Table Quality appraisal. Quality Appraisal of all included studies using the Quality Assessment for Diverse Studies checklist.
(DOCX)

S1 File Findings for tracer conditions. Findings related to the implementation and use of PROMs in Epilepsy, Heart Failure, Parkinson’s disease and Cataract surgery.
(DOCX)
