# Supplementary material for: The implementation, use and impact of patient reported outcome measures in value-based healthcare programmes: A scoping review
Source: PLoS One. 2023 Dec 6;18(12):e0290976. doi: 10.1371/journal.pone.0290976 (PMC10699630; doi:10.1371/journal.pone.0290976)
Supplement: S1 Table — Framework used for data analysis. (DOCX) [file pone.0290976.s001.docx]

# **Supporting Information 1**

# **Framework analysis used for synthesis.**

| **Framework_draft 2** | **Barriers** | **Enablers /facilitators** |
| --- | --- | --- |
| Implementation issues or observations / facilitators or barriers |  |  |
| Feasibility/acceptability |  |  |
|  | **Patients/Carers** | **Services/Healthcare professionals** |
| Costs or resources |  |  |
| Programme theory – how are the PROMs intended to work? |  |  |
| Any unintended consequences of using PROMs |  |  |
| Managing PROMs use with multiple comorbidities |  |  |
| Evidence that PROMs work? What kind of evidence is this (qual, quant, mixed, economic, what perspectives are accounted for etc etc)? |  |  |
| Differences in experiences |  |  |
| How transferable is the evidence? E.g., does this evidence depend on different healthcare systems, demographics, implementation programmes, digital infrastructure etc |  |  |
| Are there differences in experiences from different services including the focussed tracer conditions? |  |  |
| Are there any noted differences in experiences in terms of overall demographics and also in and between specific services and the tracer conditions? |  |  |
| Equity issues (digital divide, etc) |  |  |
| Are PROMs sustainable? |  |  |
| Do PROMs contribute to the NHS Post-Covid recovery plan? |  |  |
| How are PROMS implemented in the specific tracer conditions |  |  |
