# Supplementary material for: The implementation, use and impact of patient reported outcome measures in value-based healthcare programmes: A scoping review
Source: PLoS One. 2023 Dec 6;18(12):e0290976. doi: 10.1371/journal.pone.0290976 (PMC10699630; doi:10.1371/journal.pone.0290976)
Supplement: S2 Table — (DOCX) [file pone.0290976.s002.docx]

**Supporting Information 2**

**PRISMA Checklist**

S2 Table. PRISMA checklist

| **Section and Topic** | **Item #** | **Checklist item** | **Location where item is reported** |
| --- | --- | --- | --- |
| **TITLE** | | |  |
| Title | 1 | Identify the report as a systematic review.   Identified as a scoping review | Page 1, line 4 |
| **ABSTRACT** | | |  |
| Abstract | 2 | See the PRISMA 2020 for Abstracts checklist.    Adapted for a scoping review | Pages 2-3, lines 33-52 |
| **INTRODUCTION** | | |  |
| Rationale | 3 | Describe the rationale for the review in the context of existing knowledge. | Page 4, lines 76-97 |
| Objectives | 4 | Provide an explicit statement of the objective(s) or question(s) the review addresses. | Page 4, lines 95-97 |
| **METHODS** | | |  |
| Eligibility criteria | 5 | Specify the inclusion and exclusion criteria for the review and how studies were grouped for the syntheses. Adapted for a scoping review | Page 8, lines 174-179 |
| Information sources | 6 | Specify all databases, registers, websites, organisations, reference lists and other sources searched or consulted to identify studies. Specify the date when each source was last searched or consulted. Adapted for a scoping review | Page 7, lines 154-162 |
| Search strategy | 7 | Present the full search strategies for all databases, registers and websites, including any filters and limits used. Adapted for a scoping review | Page 8, lines 168-169 |
| Selection process | 8 | Specify the methods used to decide whether a study met the inclusion criteria of the review, including how many reviewers screened each record and each report retrieved, whether they worked independently, and if applicable, details of automation tools used in the process. Adapted for a scoping review | Pages 7-9, lines 158-164,180-183 |
| Data collection process | 9 | Specify the methods used to collect data from reports, including how many reviewers collected data from each report, whether they worked independently, any processes for obtaining or confirming data from study investigators, and if applicable, details of automation tools used in the process.   Adapted for a scoping review | Page 9, Lines 186-196 |
| Data items | 10a | List and define all outcomes for which data were sought. Specify whether all results that were compatible with each outcome domain in each study were sought (e.g. for all measures, time points, analyses), and if not, the methods used to decide which results to collect. Adapted for a scoping review | Pages 5-7, lines 113-152 |
|  | 10b | List and define all other variables for which data were sought (e.g. participant and intervention characteristics, funding sources). Describe any assumptions made about any missing or unclear information.  Adapted for a scoping review | Page 9, lines 192-194 |
| Study risk of bias assessment | 11 | Specify the methods used to assess risk of bias in the included studies, including details of the tool(s) used, how many reviewers assessed each study and whether they worked independently, and if applicable, details of automation tools used in the process.  Adapted for a scoping review / we performed a quality assessment | Page 10, lines 204-221 |
| Effect measures | 12 | Specify for each outcome the effect measure(s) (e.g. risk ratio, mean difference) used in the synthesis or presentation of results. Not applicable in a scoping review | N/A |
| Synthesis methods | 13a | Describe the processes used to decide which studies were eligible for each synthesis (e.g. tabulating the study intervention characteristics and comparing against the planned groups for each synthesis (item #5)).  Not applicable in a scoping review | N/A |
|  | 13b | Describe any methods required to prepare the data for presentation or synthesis, such as handling of missing summary statistics, or data conversions. Not applicable in a scoping review | N/A |
|  | 13c | Describe any methods used to tabulate or visually display results of individual studies and syntheses. Adapted for a scoping review | Page 9, lines 189-201 |
|  | 13d | Describe any methods used to synthesize results and provide a rationale for the choice(s). If meta-analysis was performed, describe the model(s), method(s) to identify the presence and extent of statistical heterogeneity, and software package(s) used. Not applicable in a scoping review | N/A |
|  | 13e | Describe any methods used to explore possible causes of heterogeneity among study results (e.g. subgroup analysis, meta-regression). Not applicable in a scoping review | N/A |
|  | 13f | Describe any sensitivity analyses conducted to assess robustness of the synthesized results. Not applicable in a scoping review | N/A |
| Reporting bias assessment | 14 | Describe any methods used to assess risk of bias due to missing results in a synthesis (arising from reporting biases). Not applicable in a scoping review | N/A |
| Certainty assessment | 15 | Describe any methods used to assess certainty (or confidence) in the body of evidence for an outcome. Not applicable in a scoping review | N/A |
| **RESULTS** | | |  |
| Study selection | 16a | Describe the results of the search and selection process, from the number of records identified in the search to the number of studies included in the review, ideally using a flow diagram. Adapted for a scoping review | Page 11, line 241 |
|  | 16b | Cite studies that might appear to meet the inclusion criteria, but which were excluded, and explain why they were excluded. | Page 11, Line 241 |
| Study characteristics | 17 | Cite each included study and present its characteristics. Adapted for a scoping review | Page 12-18 |
| Risk of bias in studies | 18 | Present assessments of risk of bias for each included study. Adapted for a scoping review / we performed a quality assessment | Page 28, lines 263-270 |
| Results of individual studies | 19 | For all outcomes, present, for each study: (a) summary statistics for each group (where appropriate) and (b) an effect estimate and its precision (e.g. confidence/credible interval), ideally using structured tables or plots. Not applicable for a scoping review | N/A |
| Results of syntheses | 20a | For each synthesis, briefly summarise the characteristics and risk of bias among contributing studies. Not applicable for a scoping review | N/A |
|  | 20b | Present results of all statistical syntheses conducted. If meta-analysis was done, present for each the summary estimate and its precision (e.g. confidence/credible interval) and measures of statistical heterogeneity. If comparing groups, describe the direction of the effect. Not applicable for a scoping review | N/A |
|  | 20c | Present results of all investigations of possible causes of heterogeneity among study results. Not applicable for a scoping review | N/A |
|  | 20d | Present results of all sensitivity analyses conducted to assess the robustness of the synthesized results. Not applicable for a scoping review | N/A |
| Reporting biases | 21 | Present assessments of risk of bias due to missing results (arising from reporting biases) for each synthesis assessed. Not applicable for a scoping review | N/A |
| Certainty of evidence | 22 | Present assessments of certainty (or confidence) in the body of evidence for each outcome assessed. Not applicable for a scoping review | N/A |
| **DISCUSSION** | | |  |
| Discussion | 23a | Provide a general interpretation of the results in the context of other evidence. Adapted for a scoping review | Pages 45-46, lines 467-502 |
|  | 23b | Discuss any limitations of the evidence included in the review. Adapted for a scoping review | Pages 48-49, lines 526-554 |
|  | 23c | Discuss any limitations of the review processes used. Adapted for a scoping review | Page 47, lines 504-543 |
|  | 23d | Discuss implications of the results for practice, policy, and future research. Adapted for a scoping review | Pages 47-48, lines 543-554 |
| **OTHER INFORMATION** | | |  |
| Registration and protocol | 24a | Provide registration information for the review, including register name and registration number, or state that the review was not registered. Adapted for a scoping review | Page 5, line 109. |
|  | 24b | Indicate where the review protocol can be accessed, or state that a protocol was not prepared. Adapted for a scoping review |  |
|  | 24c | Describe and explain any amendments to information provided at registration or in the protocol. |  |
| Support | 25 | Describe sources of financial or non-financial support for the review, and the role of the funders or sponsors in the review. | Page 50, lines 571-573 |
| Competing interests | 26 | Declare any competing interests of review authors. | Page 49, lines 567-569 |
| Availability of data, code and other materials | 27 | Report which of the following are publicly available and where they can be found: template data collection forms; data extracted from included studies; data used for all analyses; analytic code; any other materials used in the review. | Page 9, lines 190-192; & Pages 12-27. |

*From:*  Page MJ, McKenzie JE, Bossuyt PM, Boutron I, Hoffmann TC, Mulrow CD, et al. The PRISMA 2020 statement: an updated guideline for reporting systematic reviews. BMJ 2021;372:n71. doi: 10.1136/bmj.n71

For more information, visit: [http://www.prisma-statement.org/](https://eur01.safelinks.protection.outlook.com/?url=http%3A%2F%2Fwww.prisma-statement.org%2F&data=05%7C01%7Cm.silveirabianchim%40bangor.ac.uk%7C59e02e0879754495141908db47478a0f%7Cc6474c55a9234d2a9bd4ece37148dbb2%7C0%7C0%7C638182144806619719%7CUnknown%7CTWFpbGZsb3d8eyJWIjoiMC4wLjAwMDAiLCJQIjoiV2luMzIiLCJBTiI6Ik1haWwiLCJXVCI6Mn0%3D%7C3000%7C%7C%7C&sdata=QSjzur8%2BAxFUI3R2y4Pc7WT5ElaNnZLALPVw7xtfeXY%3D&reserved=0)
