# Supplementary material for: The implementation, use and impact of patient reported outcome measures in value-based healthcare programmes: A scoping review
Source: PLoS One. 2023 Dec 6;18(12):e0290976. doi: 10.1371/journal.pone.0290976 (PMC10699630; doi:10.1371/journal.pone.0290976)
Supplement: S3 Table — Characteristics of 43 studies. (DOCX) [file pone.0290976.s003.docx]

**Supporting Information 3**

**Characteristics of included studies**

**S3 Table.** Characteristics of included studies (n= 43 studies)

| **Author & year** | **Type of study** | **Country / Countries** | **Health Condition / Topic** | **Healthcare Setting** | **Population / Participants** | **Number of Participants** | **Participant Age (mean / median)** | **Sex (female %)** |
| --- | --- | --- | --- | --- | --- | --- | --- | --- |
| Allar et al (2022) | Qualitative | USA | PROMs for patients with limited English proficiency | Six academic medical centres in the Boston, MA metropolitan region | Health care providers including 21 surgeons, 2 nurse practitioners and 1 speech-language pathologist | 24 | Not reported | Not reported |
| Amini et al (2021) | Observational | Netherlands | Breast cancer  Familial hypercholesterolemia  Bladder cancer  Stroke  Brain tumours  Otolaryngology disorders  Cleft lip and palate  Turner syndrome  Paediatric sickle cell anaemia  Adult sickle cell anaemia | The Erasmus University Medical Centre, a large academic hospital | Healthcare providers and researchers involved in implementing PROMs | 61 | 46 (median) | 71 |
| Ashley et al (2013) | Feasibility | UK | Breast, Colorectal & Prostate Cancer | NHS cancer clinics in the Yorkshire area | Adults diagnosed with potentially curable breast, colorectal or prostate cancer within the last 6 months | 636 | 61.3 (mean) | 56.9 |
| Austin et al (2019) | Participatory action research | USA | Implementation of PROMs in clinical care | University of Washington (UW) Medicine, a four-hospital health system in the Seattle metropolitan area | Clinical and administrative stakeholders with an interest in PRO implementation. This included the 'UW PROs Governance Committee' members, a group charged with implementing PROMs | not reported | Not reported | not reported |
| Basch et al (2016) | Randomised Controlled Trail (RCT) | USA | Cancer: advanced solid tumours | Memorial Sloan Kettering Cancer Centre (MSK) in New York | Adults with advanced solid tumours who planned to receive chemotherapy at MSK | 766 | 61.4 (median) | 58 |
| Bernstein et al (2019) | Non-randomised trial | USA | Orthopaedic surgery | Orthopaedic surgery clinic at an academic medical centre | Adults who visited an orthopaedic surgery clinic over a 19-month period were invited to complete the research survey | 8607 | 61 (median) | 62 |
| Biber et al (2018) | Evaluative study | USA | Implementation of PROMs in clinical care | University of Utah Health which is consists of 4 hospitals & 12 community clinics | Patients visiting any of the four hospitals and 12 community clinics [400,000 unique patients per year] | not reported | not reported | not reported |
| Clary et al (2022) | Pragmatic Randomised Controlled trial (RCT) | USA | Epilepsy | Tertiary adult epilepsy clinic in the South-eastern United Sates | Patients with a diagnosis of epilepsy and high or borderline anxiety or depression symptoms based on electronic response to anxiety and depression instruments | 30 | 42.8 | 60 |
| Damman et al (2019) | Mixed methods study (qualitative + quantitative) | Netherlands | Parkinson's Disease | Not conducted within a healthcare setting | Healthcare providers who participated in interview study (n = 14)  Adults with Parkinson's Disease who participated in interview study (n = 13)  Adults with Parkinson's Disease who participated in survey study (n = 113) | 140 | Not reported | 70.7 |
| Demedts et al (2021) | Quasi-experimental case-control | Belgium | Lung Cancer | A hospital oncology day clinic | Stage IV lung cancer patients and earlier stage patients added later on in the trial | 221 | 65.9 (mean) | 24 |
| Devlin et al (2010) | Pilot | UK | Four surgical procedures: cataract surgery, varicose vein surgery, knee replacement, & hip replacement | 24 healthcare providers: 7 NHS treatment centres, 13 NHS acute hospitals, 2 independent sector hospitals, 1 private hospital treating NHS funded patients, and 1 NHS general practice (for hernia repair only) | Patients having cataract, varicose vein, knee replacement or hip replacement surgery | 2407 | Not reported | Not reported |
| Fung et al (2016) | Prospective cohort study | UK | Cataract surgery | Moorfields Eye Hospital, London | Patients having cataract surgery for one or both eyes | 122 | 70.7 | 48 |
| Goretti et al (2020) | Feasibility study | Italy | Bariatric surgery | Specialised academic hospital in northern Italy | Morbidly obese patients undergoing gastric sleeve or gastric bypass surgery | 2122 | 42 | 70 |
| Groeneveld et al (2019) | Observational study | Netherlands | Stroke | An inpatient and outpatient stroke rehabilitation centre | Patients admitted to the inpatient or outpatient rehabilitation centre in the first year after a stroke | 373 | 59.7 (mean) | 41.6 |
| Kane & Daveson et al (2017) | Mixed methods feasibility and acceptability study | Ireland | Chronic heart failure | Nurse-led chronic heart failure disease management clinics in two national tertiary referral centres in Dublin | Patients attending chronic heart failure management clinic with a diagnosis of chronic heart failure | 25 | 75.7 | 43.5 |
| Kane & Ellis-smith et al (2017) | Qualitative | Ireland | Chronic heart failure | Nurse-led chronic heart failure disease management clinics in two national tertiary referral centres in Dublin | Patients attending chronic heart failure management clinic with a diagnosis of chronic heart failure | 18 | 75 | 39 |
| Laureij et al (2020) | Mixed methods clinical study | Netherlands | Pregnancy & childbirth | Five obstetric collaborative networks (OCNs) consisting of several primary care midwifery practices and maternal care organisations and at least one tertiary level hospital | Pregnant patients (n = 142), health care professionals (n = 134) and administrators (n = 35) | 311 | 33  (this includes patient participant only of which n = 142) | 100  (this includes patient participant only of which n = 142) |
| Liu et al (2018) | Qualitative study | USA | Hip and knee pain | Arthroplasty clinic | Patients with knee and hip pain | 51 | 57 | 57 |
| Malhotra et al (2016) | Prospective cohort study | UK | Orthopaedic surgery | Specialist surgical orthopaedic clinic in an NHS hospital | All new adult patients attending elective orthopaedic outpatient clinics over a 32-month period | 2176 | 21.76 | 59.88 |
| Moura & Magliocco et al (2016) | Feasibility study | USA | Epilepsy | Ambulatory neurology clinic | All outpatient adults who attended a neurology clinic for epilepsy | 6075 | 56.5 | 54.11 |
| Moura & Schwamm et al (2019) | Cross-sectional & longitudinal | USA | Epilepsy | Ambulatory neurology clinic | All adult patients with an appointment in the epilepsy clinic | 610 | 42.2 | 47.2 |
| Nguyen et al (2018) | Prospective cohort | Belgium | Locally Advanced Non-Small Cell Lung Cancer | Single centre oncology clinic | Patients diagnosed with locally advanced non-small cell lung cancer (TNM7) | 32 | 62 | 25 |
| O'Connell et al (2018) | Feasibility study | UK | Wide range of conditions in primary and secondary care | NHS, Wales health boards:   - Ysbyty Gwynedd, Bangor - Betsi Cadwaladr UHB - Aneurin Bevan UHB - Cardiff and Vale University Health Board - Hywel Dda UHB - Abertawe Bro Morgannwg UHB - Cwm Taf UHB | NHS secondary care patients | 5366 | Not reported | Not reported |
| Oemrawsingh et al (2019) | Observational study | Netherlands | Ischemic stroke patients | Adults diagnosed with ischemic stroke | Four stroke care centres in the Netherlands, 1 academic hospital, and 3 district-based hospitals. | 1022 | 74 | Not reported |
| Papuga et al (2018) | Implementation study (evaluative) | USA | Orthopaedic conditions | Department of Orthopaedic Surgery and Rehabilitation in a large academic hospital | Adult patients who attended the orthopaedic clinic | 17892 | Not reported | Not reported |
| Pennucci et al (2020) | Mixed methods pilot study | Italy | Chronic heart failure | Fondazione Toscana Gabriele Monasterio (FTGM), a tertiary referral centre for chronic heart failure. | Adults hospitalised for chronic heart failure | 162 | 73.0 | 29.6 |
| Peters et al (2013) | Mixed methods pilot study | UK | Asthma, chronic obstructive pulmonary disease (COPD), diabetes, epilepsy, heart failure & stroke | 33 primary care practices in London (n=18) and the North-West of England (n=15) | Adults diagnosed with asthma, COPD, diabetes, epilepsy, heart failure or stroke | 1721 | Not reported | 47.4 |
| Peters et al (2014) | Cohort study | UK | Asthma, chronic obstructive pulmonary disease (COPD), diabetes, epilepsy, heart failure & stroke | 33 primary care practices in London (n=18) and the North-West of England (n=15) | Adults diagnosed with asthma, COPD, diabetes, epilepsy, heart failure or stroke | 1721 | Not reported | 47.4 |
| Peters & Croker et al (2014) | Cohort study | UK | Asthma, chronic obstructive pulmonary disease (COPD), diabetes, epilepsy, heart failure & stroke | 33 primary care practices in London (n=18) and the North-West of England (n=15) | Adults diagnosed with asthma, COPD, diabetes, epilepsy, heart failure or stroke | 1721 | Not reported | 47.4 |
| Porter et al (2021) | Feasibility study | UK | Asthma, chronic obstructive pulmonary disease (COPD), diabetes, heart failure, depression, & hip/knee osteoarthritis | General Practice, NHS | Patients with two or more highly prevalent conditions (asthma, COPD, diabetes, heart failure, depression, and hip/knee osteoarthritis) | 68 | 70 | 47 |
| Queirós et al (2021) | Retrospective observational study | Portugal | All patients submitted to cataract surgery at IPO-Porto, with 3 months follow up period | Cataract surgery at the Portuguese Institute of oncology | All patients submitted to cataract surgery at IPO-Porto, with 3 months follow up period | 268 | 73 | 62.8 |
| Rutherford et al (2021) | Mixed-methods evaluative study | Australia | Broad range of chronic conditions including heart disease, skin cancer, diabetes, chronic obstructive pulmonary disease (COPD), back pain, osteoarthritis, and alcohol and other drugs | 18 sites across New South Wales | Patients with chronic conditions | 1884 | 72.7 | 47.4 |
| Sajobi et al (2021) | Cohort study | Canada | Epilepsy | Foothills Medical Centre, a tertiary care centre in Calgary, Alberta | Adults with epilepsy seen at the Foothills Medical Centre, a tertiary care centre in Calgary, Alberta. Participants are part of the Calgary Comprehensive Epilepsy Program - a prospective registry of patients with epilepsy in Calgary, Alberta | 1143 | 37 | 29.6 |
| Schuler et al (2017) | Cross-sectional | Germany | Cancer | An inpatient cancer care unit in a large German University Hospital | Adults diagnosed with cancer admitted to the cancer department | 192 | 53.8 | 44.3 |
| Sparrow et al (2018) | Psychometric study | UK | Cataract surgery | Four cataract surgical centres in England (Bristol, Torbay, Cheltenham, Brighton) | Adults having cataract surgery for one or both eyes | 822 | 76 | 58 |
| Sparrow et al (2018) | Feasibility study | UK | Cataract surgery | Ophthalmology surgery department in an NHS hospital | Adults having cataract surgery for one or both eyes | 207 | 78 | 49 |
| Tognetto et al (2021) | Prospective observational study | Italy | Cataract surgery | University Eye Clinic of Trieste, Italy | Adults with a  diagnosis of operable cataract | 218 | 74 | 64.2 |
| van der Wilik et al (2019) | Mixed method developmental study | Netherlands | Chronic kidney disease (CKD) | Not provided | Adults with a diagnosis of chronic kidney disease | 151 | 60.4 | Not provided |
| van Egdom et al (2019) | Implementation and evaluative report | Netherlands | Breast cancer | The Erasmus MC Cancer Institute, a major academic healthcare institute which has initiated a VBHC-strategy. The institute has an integrated multidisciplinary breast cancer practice unit | Adults diagnosed with breast cancer | 239 | Not provided | 100 |
| Wheelock et al (2014) | Randomised Controlled Trail (RCT) | USA | Breast Cancer | University of California at San Francisco’s Breast Care Centre | Patients with TNM stage I to III breast cancer | 102 | 52.9 | 100 |
| Withers et al (2020) | Development and evaluative study | UK | NHS Wales PROMs implementation | All Health Boards in Wales, the NHS Wales Informatics Service (NWIS), and Cedar (a healthcare technology research centre) | Not reported | Not reported | Not reported | Not reported |
| Zijlmans et al (2021) | Prospective non-interventional observational descriptive study | Netherlands, Germany, UK, Italy, Spain & Finland | Cataract surgery | 10 European health institutions / hospitals in Finland (n =1), Spain (n = 4), The Netherlands (n=2), Germany (n = 1), UK (n = 1), Italy (n = 1) | Patients having cataract surgery for one or both eyes | 3799 | 72.7 | 56.3 |
