# Supplementary material for: The implementation, use and impact of patient reported outcome measures in value-based healthcare programmes: A scoping review
Source: PLoS One. 2023 Dec 6;18(12):e0290976. doi: 10.1371/journal.pone.0290976 (PMC10699630; doi:10.1371/journal.pone.0290976)
Supplement: S4 Table — Quality Appraisal of all included studies using the Quality Assessment for Diverse Studies checklist. (DOCX) [file pone.0290976.s004.docx]

**Supporting Information 4**

**Quality Appraisal**

**S4 Table.** Quality Appraisal of all included studies using the Quality Assessment for Diverse Studies checklist (n = 43 studies)

| **Author & Year** | **Theoretical or conceptual underpinning to the research** | **Statement of research aim / s** | **Clear description of research setting and target population** | **The study design is appropriate to address the stated research aim /s** | **Appropriate sample to address the research aim /s** | **Rationale for choice of data collection tool /s** | **The format and content of data collection tool is appropriate to address the stated research aim /s** | **Description of data collection procedure** | **Recruitment data provided** | **Justification for analytic method selected** | **The method of analysis was appropriate to answer the research aim /s** | **Evidence that the research stakeholders have been considered in research design or conduct** | **Strengths and limitations critically discussed** | **Final Rating** |
| --- | --- | --- | --- | --- | --- | --- | --- | --- | --- | --- | --- | --- | --- | --- |
| Allar et al, 2022 | 3 | 3 | 3 | 3 | 3 | 3 | 3 | 3 | 2 | 3 | 3 | 2 | 3 | No to minor methodological concerns |
| Amini et al, 2021 | 2 | 3 | 2 | 2 | 2 | 2 | 1 | 2 | 2 | 1 | 2 | 1 | 3 | Moderate methodological concerns |
| Ashley et al, 2013 | 3 | 3 | 3 | 3 | 2 | 3 | 3 | 3 | 3 | 3 | 3 | 1 | 3 | No/minor methodological concerns |
| Austin et al, 2019 | 2 | 1 | 2 | 3 | 1 | 3 | 3 | 2 | 1 | 3 | 3 | 3 | 3 | No/minor methodological concerns |
| Basch et al, 2016 | 3 | 3 | 3 | 3 | 2 | 3 | 3 | 3 | 3 | 3 | 3 | 0 | 3 | No/minor methodological concerns |
| Bernstein et al, 2019 | 3 | 3 | 2 | 1 | 0 | 1 | 2 | 1 | 0 | 3 | 3 | 0 | 2 | Serious methodological concerns |
| Biber et al, 2018 | 2 | 2 | 0 | 1 | 1 | 0 | 1 | 0 | 0 | 0 | 1 | 1 | 0 | Serious methodological concerns |
| Clary et al, 2022 | 1 | 3 | 3 | 3 | 3 | 3 | 3 | 3 | 3 | 2 | 3 | 0 | 3 | No/minor methodological concerns |
| Damman et al, 2019 | 1 | 3 | 3 | 3 | 3 | 2 | 1 | 2 | 2 | 1 | 3 | 2 | 3 | Moderate methodological concerns |
| Demedts et al, 2021 | 2 | 3 | 1 | 3 | 3 | 1 | 2 | 2 | 2 | 0 | 2 | 0 | 3 | Moderate methodological concerns |
| Devlin et al, 2010 | 3 | 3 | 3 | 2 | 3 | 3 | 2 | 1 | 2 | 2 | 2 | 0 | 3 | No/minor methodological concerns |
| Fung et al, 2016 | 2 | 3 | 3 | 3 | 2 | 3 | 3 | 3 | 3 | 3 | 3 | 0 | 3 | No/minor methodological concerns |
| Gorretti et al, 2020 | 3 | 3 | 3 | 2 | 3 | 2 | 3 | 3 | 2 | 1 | 3 | 2 | 1 | No/minor methodological concerns |
| Groeneveld et al, 2019 | 3 | 3 | 2 | 3 | 3 | 3 | 3 | 3 | 3 | 3 | 3 | 1 | 3 | No/minor methodological concerns |
| Kane & Daveson et al et al, 2017 | 1 | 3 | 3 | 3 | 3 | 3 | 3 | 3 | 3 | 0 | 3 | 0 | 2 | No/minor methodological concerns |
| Kane & Ellis-smith et al et al, 2017 | 1 | 3 | 3 | 3 | 3 | 3 | 3 | 3 | 3 | 2 | 3 | 0 | 3 | No/minor methodological concerns |
| Laureiji et al, 2020 | 1 | 3 | 3 | 3 | 3 | 2 | 3 | 3 | 3 | 2 | 3 | 0 | 3 | No/minor methodological concerns |
| Liu et al, 2017 | 1 | 3 | 3 | 3 | 3 | 3 | 3 | 3 | 3 | 2 | 3 | 0 | 3 | No/minor methodological concerns |
| Malhotra et al, 2016 | 2 | 3 | 3 | 3 | 3 | 3 | 3 | 3 | 3 | 2 | 3 | 0 | 3 | No/minor methodological concerns |
| Moura & Magliocco et al, 2019 | 1 | 3 | 2 | 3 | 0 | 2 | 3 | 3 | 3 | 2 | 2 | 0 | 3 | No/minor methodological concerns |
| Moura & Schwann et al, 2016 | 2 | 3 | 2 | 3 | 3 | 3 | 3 | 3 | 2 | 3 | 3 | 0 | 3 | No/minor methodological concerns |
| Nguyen et al, 2018 | 1 | 2 | 2 | 2 | 3 | 3 | 2 | 3 | 1 | 3 | 1 | 0 | 1 | Moderate methodological concerns |
| O'Connell et al, 2018 | 3 | 3 | 3 | 3 | 2 | 3 | 3 | 3 | 1 | 3 | 3 | 2 | 0 | No/minor methodological concerns |
| Omerawingh et al, 2019 | 1 | 2 | 3 | 3 | 2 | 2 | 3 | 2 | 2 | 2 | 3 | 0 | 3 | No/minor methodological concerns |
| Papuga et al, 2019 | 3 | 3 | 3 | 3 | 0 | 3 | 3 | 1 | 0 | 3 | 3 | 3 | 1 | No/minor methodological concerns |
| Pennuci et al, 2020 | 1 | 1 | 2 | 3 | 2 | 3 | 3 | 3 | 3 | 3 | 3 | 3 | 0 | No/minor methodological concerns |
| Peters et al, 2013 | 3 | 3 | 3 | 3 | 3 | 3 | 3 | 3 | 3 | 3 | 3 | 2 | 1 | No/minor methodological concerns |
| Peters, Crockett & Jenkinson et al, 2014 | 1 | 3 | 3 | 3 | 3 | 3 | 3 | 2 | 2 | 2 | 2 | 1 | 2 | No/minor methodological concerns |
| Peters, Crockett & Dummett et al, 2021 | 2 | 2 | 2 | 2 | 3 | 2 | 2 | 3 | 2 | 2 | 2 | 1 | 2 | No/minor methodological concerns |
| Porter et al, 2021 | 3 | 3 | 2 | 2 | 0 | 1 | 1 | 3 | 3 | 3 | 2 | 2 | 2 | Serious concerns methodological concerns |
| Querios et al, 2021 | 2 | 3 | 3 | 3 | 3 | 3 | 3 | 3 | 3 | 3 | 3 | 2 | 3 | No/minor methodological concerns |
| Rutherford et al, 2020 | 2 | 3 | 3 | 3 | 3 | 2 | 3 | 3 | 3 | 3 | 2 | 3 | 3 | No/minor methodological concerns |
| Sajobi et al, 2021 | 2 | 3 | 3 | 3 | 3 | 3 | 3 | 3 | 3 | 3 | 3 | 1 | 3 | No/minor methodological concerns |
| Schuler et al, 2017 | 2 | 3 | 3 | 3 | 3 | 3 | 3 | 3 | 3 | 3 | 3 | 0 | 3 | No/minor methodological concerns |
| Sparrow et al, 2020 | 2 | 3 | 3 | 3 | 3 | 3 | 3 | 3 | 3 | 3 | 3 | 0 | 3 | No/minor methodological concerns |
| Sparrow et al, 2018 | 2 | 3 | 3 | 3 | 2 | 3 | 3 | 3 | 2 | 2 | 2 | 2 | 2 | Moderate methodological concerns |
| Tognetto et al, (2021) | 2 | 3 | 3 | 3 | 3 | 3 | 3 | 1 | 3 | 3 | 3 | 0 | 0 | No/minor methodological concerns) |
| Van der Wilik et al, 2018 | 2 | 3 | 3 | 3 | 3 | 3 | 3 | 3 | 3 | 3 | 3 | 3 | 3 | No/minor methodological concerns |
| Van Egdom et al, 2019 | 2 | 3 | 3 | 3 | 3 | 3 | 3 | 3 | 3 | 3 | 3 | 3 | 3 | No/minor methodological concerns |
| Wheelock et al, 2014 | 3 | 3 | 3 | 3 | 3 | 3 | 3 | 3 | 3 | 3 | 3 | 0 | 3 | No/minor methodological concerns |
| Withers et al, 2020 | 3 | 3 | 3 | 3 | 3 | 3 | 3 | 2 | 0 | 2 | 2 | 3 | 3 | Moderate methodological concerns |
| Zijlmans et al, 2021 | 3 | 3 | 3 | 3 | 3 | 3 | 3 | 2 | 2 | 3 | 2 | 1 | 3 | No/minor methodological concerns |

0 – No/minor methodological concerns; 1 - Moderate methodological concerns; 2 - Serious concerns methodological concerns; 3 – Very serious concerns methodological concerns
