# Supplementary material for: The implementation, use and impact of patient reported outcome measures in value-based healthcare programmes: A scoping review
Source: PLoS One. 2023 Dec 6;18(12):e0290976. doi: 10.1371/journal.pone.0290976 (PMC10699630; doi:10.1371/journal.pone.0290976)
Supplement: S1 File — Findings related to the implementation and use of PROMs in Epilepsy, Heart Failure, Parkinson’s disease and Cataract surgery. (DOCX) [file pone.0290976.s005.docx]

# **Supporting Information 5**

# **Findings related to the implementation and use of PROMS in the specific tracer conditions**

### **Parkinson’s disease**

There was a lack of evidence in Parkinson’s disease with only one study investigating the use of PROMs for this condition, including a total of 128 patients with Parkinson’s disease [1,2].

Damman [2] conducted semi‐structured interviews with 13 patients with Parkinson’s disease and with 7 neurologists and 7 physiotherapists to understand how patients and medical professionals think about PROMs during routine medical consultations. The authors also conducted a survey on 115 patients. Overall, healthcare professionals preferred the use of individual PROMs data over time to monitor disease progression and treatment efficiency. They also described how PROMs data could be used to facilitate patient-centred care and shared decision making. However, the authors highlighted conflicting expectations regarding who should initiate a conversation about PROMs during the consultation. While healthcare professionals expected patients to initiate a discussion about PROMs, patients stated not feeling equipped to initiate a conversation about it on their own. In general, patients mentioned that PROMs data were not discussed during consultations and healthcare professionals expressed feeling the need to receive training on how to use PROMs during appointments with patients.

### **Heart Failure**

Three studies investigating the use of PROMs in heart failure, included a total of 147 patients and 4 healthcare professionals [3-5].

Kane [3] interviewed 18 patients and 4 nurses to understand whether and how a palliative-specific PROM-based intervention facilitated patients’ experience of disease management. Patients, including two with self-acknowledged suboptimal literacy, described PROMs delivery as user friendly and easy to read. This is a significant finding given the increasing age, cognitive impairment and comorbidities associated with heart failure. Kane & Daveson [4] also demonstrated the feasibility and acceptability of a PROM intervention in 25 heart failure patients. The intervention used a palliative care outcome scale and trained nurses to manage symptoms and concerns identified by heart failure patients using PROMs. PROMs helped to identify issues in usual care such as psychological distress and financial needs. Whilst the authors were unable to provide empirical evidence regarding the efficacy of PROMs, the study demonstrated the feasibility and acceptability of using this type of intervention in heart failure.

Pennucci [5] performed a pilot to investigate the implementation of a specific design for assessing chronic heart failure patients clinical’ pathway. The intervention was designed to collect longitudinal PROMs data, administering four questionnaires per each enrolled patient and included 104 (64%) patients at baseline, 95 (61%) at 1 month, 41 (49%) at 7 months and 9 (31%) at 12 months. Measures of generic and specific PROMs along with experience of care and self-care dimensions were collected. Patients received their first online questionnaire via a personal link sent to patients or caregivers by phone or email, according to their preference. Patients’ answers were collected in real time and data were longitudinally reported on an online platform resulting in improved organisational processes and interprofessional coordination. PROMs also helped to improve the identification of unrecognised problems which was particularly helpful in chronic heart failure patients who typically under report their high symptoms burden. For example, PROMs enabled the identification of unmet needs such as psychosocial concerns which were not being addressed until end-of-life care. Nurses acknowledged that during a busy clinic they often did not have the time to investigate these issues.

### **Epilepsy**

Two studies investigating the use of PROMs in epilepsy, included a total of 6,680 patients [6-8].

Moura [6] demonstrated the feasibility of administering online surveys to collect PROMs in an ambulatory neurology clinic over a period of 3 months. The study successfully collected PROMs from 2,992 patients, among these 520 had epilepsy. The authors concluded that feasibility relied heavily on administrative and care provider staff training and support. Moura [7] performed a secondary analysis on data from 160 patients as part of an ongoing improvement project in an academic, tertiary referral epilepsy clinic. The authors used the Patient Reported Outcomes Measurement Information System (PROMIS) to assess health metrics within and across epilepsy. PROMs questionnaires were integrated with the clinic and all patients had a chance to discuss it with their physician during appointments. Overall, the use of PROMs highlighted continued seizures, side effects and cost of medication as areas of need for intervention.

### **Heart Failure & Epilepsy**

Peter’s [8], Peters, Crocker & Dummett [9] and Peters, Crocker & Jenkinson [10] conducted a cohort study involving 33 primary practices and 4,485 patients with long-term health conditions, including heart failure and epilepsyClick or tap here to enter text.. Patients were sent a generic PROM (EQ-5D) and a disease specific PROM at baseline and at follow-up after 1 year. The majority of stakeholders expressed positive views regarding the collection of PROMs data in primary care suggesting that it is feasible to implement PROMs in general practices. Despite that, further work is warranted to enhance feasibility as the logistics of this intervention were found to be too complicated. For instance, some concerns were raised regarding resources and benefits. For example, this study used postal PROMs which increase the use of resources and demands on staff.

### **Cataract Surgery**

Seven studies investigating the use of PROMs in cataract surgery pathways, included a total of 6,120 patients [11-18].

Sparrow [11] adapted a software system that is used in cataract surgery (Medisoft Electronic Medical Record [EMR]) to include the electronic capture of Cat-PROM5 pre- and post-operatively. The implementation of this new system was only successful in the pilot centre which raises concerns regarding its feasibility. Challenges were identified during the piloting and were described in detail in the ‘barriers and facilitators’ section.

Sparrow [12] demonstrated that participants preferred the Cat-PROM5 in comparison to the Catquest-9SF, as it enquired about individual vision-related factors, although both questionnaires were well received. A longitudinal study by Fung et al [13] compared different PROMs for cataract patients in the UK and demonstrated that both Catquest-9SF and VF-8R were highly sensitive to changes in visual function and quality of life, with Catquest-9SF being the most responsive measuring tool. Improvements in patient-reported visual function could be detected as early as 3 weeks post-operatively [13,14]. In contrast, generic PROMs (i.e., EQ-5D and EQ-VAS) produced insufficient response to cataract surgery and current evidence suggests they should not be used for cataract patients [13,15]. This is consistent with conflicting reports regarding the validity and sensitivity of EQ-5D in patients with visual or ophthalmic related conditions [13-15].

The incorporation of PROMs into routine practice had the potential to assist clinical decision-making and help to assess the value of ophthalmic interventions. In terms of outcomes, visual acuity measurements correlated poorly with patient reported visual function, indicating that visual acuity does not fully reflect patients’ visual function. This highlights the relevancy of using PROMs, whilst also considering clinically reported measurements when evaluating the outcomes of cataract surgery [13,14]. Evidence suggests that cataract surgery is associated with marked improvements in vision-related activity limitation and satisfaction with vision and resulted in significant improvement in PROMs [13,16,17].

PROMs increased patient engagement, allowed a better assessment of the results of cataract surgery for clinicians, provided a good basis for comparisons with other institutions nationally (Portugal) and globally, helped the clinical health team to better understand and value the effects of surgery on the quality of life of these patients [18].

# **References**

1. de Roos P, Bloem BR, Kelley TA, Antonini A, Dodel R, Hagell P, et al. A Consensus Set of Outcomes for Parkinson’s Disease from the International Consortium for Health Outcomes Measurement. J Parkinsons Dis [Internet]. 2017 [cited 2022 Dec 8];7(3):533–43. Available from: <https://pubmed.ncbi.nlm.nih.gov/28671140/>
2. Damman OC, Verbiest MEA, Vonk SI, Berendse HW, Bloem BR, de Bruijne MC, et al. Using PROMs during routine medical consultations: The perspectives of people with Parkinson’s disease and their health professionals. Health Expect [Internet]. 2019 Oct 1 [cited 2022 Dec 8];22(5):939–51. Available from: <https://pubmed.ncbi.nlm.nih.gov/31199574/>
3. Kane PM, Ellis-Smith CI, Daveson BA, Ryan K, Mahon NG, McAdam B, et al. Understanding how a palliative-specific patient-reported outcome intervention works to facilitate patient-centred care in advanced heart failure: A qualitative study. Palliat Med [Internet]. 2018 Jan 1 [cited 2022 Dec 8];32(1):143–55. Available from: <https://pubmed.ncbi.nlm.nih.gov/29154724/>
4. Kane PM, Daveson BA, Ryan K, Ellis-Smith CI, Mahon NG, McAdam B, et al. Feasibility and acceptability of a patient-reported outcome intervention in chronic heart failure. BMJ Support Palliat Care [Internet]. 2017 Dec 1 [cited 2022 Dec 8];7(4):470–9. Available from: <https://pubmed.ncbi.nlm.nih.gov/28864449/>
5. Pennucci F, de Rosis S, Passino C. Piloting a web-based systematic collection and reporting of patient-reported outcome measures and patient-reported experience measures in chronic heart failure. BMJ Open [Internet]. 2020 Oct 5 [cited 2022 Dec 8];10(10). Available from: https://pubmed.ncbi.nlm.nih.gov/33020096/
6. Moura LMVR, Schwamm E, Moura Junior V, Seitz MP, Hsu J, Cole AJ, et al. Feasibility of the collection of patient-reported outcomes in an ambulatory neurology clinic. Neurology [Internet]. 2016 Dec 6 [cited 2022 Dec 8];87(23):2435–42. Available from: https://pubmed.ncbi.nlm.nih.gov/27815405/
7. Moura LMVR, Magliocco B, Ney JP, Cheng EM, Esper GJ, Hoch DB. Implementation of quality measures and patient-reported outcomes in an epilepsy clinic. Neurology [Internet]. 2019 Nov 26 [cited 2022 Dec 8];93(22):E2032–41. Available from: https://pubmed.ncbi.nlm.nih.gov/31666351/
8. Peters M, Crocker H, Dummett S, Jenkinson C, Doll H, Gibbons E, et al. Pilot study of patient reported outcome measures (PROMs) in primary care Report to the Department of Health. 2013;
9. Peters M, Crocker H, Jenkinson C, Doll H, Fitzpatrick R. The routine collection of patient-reported outcome measures (PROMs) for long-term conditions in primary care: a cohort survey. BMJ Open [Internet]. 2014 [cited 2022 Dec 8];4(2). Available from: <https://pubmed.ncbi.nlm.nih.gov/24561495/>
10. Peters M, Crocker H, Dummett S, Jenkinson C, Doll H, Fitzpatrick R. Change in health status in long-term conditions over a one year period: A cohort survey using patient-reported outcome measures. Health Qual Life Outcomes [Internet]. 2014 Aug 12 [cited 2022 Dec 8];12(1):1–10. Available from: <https://hqlo.biomedcentral.com/articles/10.1186/s12955-014-0123-2>
11. Sparrow JM. PROMs in Cataract Surgery: The feasibility of electronic auditing of self-reported outcomes using Cat-PROM5 Patient Reported Outcomes Measures in Cataract Surgery: The feasibility of electronically auditing self-reported Outcomes using Cat-PROM5. 2020 [cited 2022 Dec 8]; Available from: www.hqip.org.uk/national-programmes.
12. Sparrow JM, Grzeda MT, Frost NA, Johnston RL, Liu CSC, Edwards L, et al. Cataract surgery patient-reported outcome measures: a head-to-head comparison of the psychometric performance and patient acceptability of the Cat-PROM5 and Catquest-9SF self-report questionnaires. Eye (Lond) [Internet]. 2018 Apr 1 [cited 2022 Dec 8];32(4):788–95. Available from: https://pubmed.ncbi.nlm.nih.gov/29386619/
13. Fung SSM, Luis J, Hussain B, Bunce C, Hingorani M, Hancox J. Patient-reported outcome measuring tools in cataract surgery: Clinical comparison at a tertiary hospital. J Cataract Refract Surg [Internet]. 2016 Dec 1 [cited 2022 Dec 8];42(12):1759–67. Available from: https://pubmed.ncbi.nlm.nih.gov/28007107/
14. Zijlmans BL, van Zijderveld R, Manzulli M, Garay-Aramburu G, Czapski P, Eter N, et al. Global multi-site, prospective analysis of cataract surgery outcomes following ICHOM standards: the European CAT-Community. Graefes Arch Clin Exp Ophthalmol [Internet]. 2021 Jul 1 [cited 2022 Dec 8];259(7):1897–905. Available from: https://pubmed.ncbi.nlm.nih.gov/33855602/
15. Devlin NJ, Parkin D, Browne J. Patient-reported outcome measures in the NHS: new methods for analysing and reporting EQ-5D data. Health Econ [Internet]. 2010 [cited 2022 Dec 8];19(8):886–905. Available from: https://pubmed.ncbi.nlm.nih.gov/20623685/
16. Queirós L, Redondo P, França M, Silva SE, Borges P, de Melo AB, et al. Implementing ICHOM standard set for cataract surgery at IPO-Porto (Portugal): clinical outcomes, quality of life and costs. BMC Ophthalmol [Internet]. 2021 Dec 1 [cited 2022 Dec 8];21(1):1–10. Available from: https://bmcophthalmol.biomedcentral.com/articles/10.1186/s12886-021-01887-6
17. Tognetto D, Giglio R, de Giacinto C, Dell’Aquila C, Pian G, Scardellato C, et al. Cataract standard set for outcome measures: An Italian tertiary referral centre experience. Eur J Ophthalmol [Internet]. 2021 Mar 1 [cited 2022 Dec 8];32(2):902–10. Available from: https://pubmed.ncbi.nlm.nih.gov/34053333/
18. Queirós L, Redondo P, França M, Silva SE, Borges P, de Melo AB, et al. Implementing ICHOM standard set for cataract surgery at IPO-Porto (Portugal): clinical outcomes, quality of life and costs. BMC Ophthalmol [Internet]. 2021 Dec 1 [cited 2022 Dec 8];21(1):1–10. Available from: https://bmcophthalmol.biomedcentral.com/articles/10.1186/s12886-021-01887-6
